# Artificial intelligence assisted multi-model pathological diagnosis of breast cancer based on multispectral autofluorescence images

**DOI:** 10.1038/s41523-026-00915-2

**Published:** 2026-03-12

**Authors:** Jiahong Sun, Jianqiao Ye, Siyi Chen, Zitong Yang, Ge Xu, Yuanbo Xue, Zi Ou, Xingye Chen, Jiandong Wang

**Affiliations:** 1 Senior Department of General Surgery, Chinese PLA General Hospital, Beijing, China; 2 Medical School of Chinese PLA, Beijing, China; 3 School of Electronic and Information Engineering, Beihang University, Beijing, China

**Keywords:** Cancer, Computational biology and bioinformatics, Oncology

## Abstract

Virtual staining technology offers a promising solution to overcome the time-consuming and sample-consumption nature of conventional histochemical staining in breast cancer pathology. This study presents a novel framework integrating multispectral autofluorescence imaging with an optimized deep learning architecture to generate high-fidelity, label-free, hematoxylin and eosin-equivalent images. We constructed a multimodal database containing clinical specimens, mouse models, and organoid co-cultures. By enhancing CycleGAN with saliency and global feature consistency losses, multispectral autofluorescence imaging-to-H&E virtual staining performance was significantly improved. This framework learns from unpaired datasets, eliminating the need for pixel-level registration. In blinded evaluations by five board-certified pathologists, 82.2% of virtual staining images achieved clinical scores comparable to conventional staining, with no statistical differences in key diagnostic indices. Moreover, this approach is non-destructive—the same tissue section remains intact for subsequent assays such as single-nucleus RNA sequencing or spatial transcriptomics, maximizing the utility of precious biopsy samples. In summary, this robust framework enables the rapid, non-destructive generation of diagnostic-grade breast cancer pathological images, making it a potential tool for clinical diagnostics and mechanistic studies across diverse biological systems.

## Introduction

Breast cancer remains a major global public health challenge, standing as the most commonly diagnosed malignancy in women and the second most prevalent cancer worldwide^1^. The 2022 Global Cancer Burden Report underscores that breast cancer accounts for approximately 25% of all new female cancer cases and 16% of all female cancer-related deaths^2^. In China, the disease burden is equally substantial: recent national statistics document 357,000 new breast cancer cases (representing 23.8% of all new female cancer diagnoses) and 75,000 deaths (accounting for 15.4% of female cancer mortality)^1^. Notably, the incidence of breast cancer among Chinese women aged ≤35 years has increased from 4.0% in 2000 to 5.9% in 2017, with an average annual growth rate of 2% and a simultaneous decline in the median age of onset^3,4^.

Pathological diagnosis, recognized as the gold standard for breast cancer management and clinical decision-making, underpins disease confirmation, prognostic stratification, and targeted therapeutic strategy formulation by systematically analyzing cellular morphology, tissue architecture disruption, and molecular marker expression patterns in histopathological sections^5–7^. Given the inherent transparency of native tissue sections, histological staining is indispensable for enhancing contrast, which serves as a prerequisite for standardizing samples in microscopic histopathological research^8^. Hematoxylin and Eosin (H&E) staining, the most widely used histological technique, generates high nuclear-cytoplasmic contrast and enables effective preservation of critical diagnostic features^9,10^. However, conventional H&E staining suffers from some limitations. First, it is plagued by inefficiency and variability. The multi-step workflow, which encompasses fixation, dehydration, embedding, sectioning, and staining, requires 24–48 h to complete, and staining quality remains highly dependent on operator expertise^11,12^. Second, during the preparation of pathological sections, the conventional workflow typically integrates formalin-fixed paraffin-embedded (FFPE) processing with H&E staining, which induces irreversible modifications to the specimen. The chemical reactions between staining agents and tissue components, coupled with structural damage triggered by FFPE processing, substantially compromise the utility of the specimen for downstream multi-omics analyses^13^.

Autofluorescence imaging (AFI) is a non-invasive, label-free technique that enables the monitoring of tissue structure and function by exciting the specific emission spectra of endogenous fluorophores in biological tissues^14^. Alterations in cellular morphology, transitions in metabolic state, and perturbations in the microenvironment markedly modify the abundance, spatial distribution, and photophysical properties of these fluorophores. Their distinct spectral features serve as intrinsic biomarkers for tissue physiological and pathological states, thereby providing a molecular foundation for disease diagnosis^15^. For instance, hyperspectral AFI facilitates the non-invasive screening of Alzheimer’s disease by resolving the specific spectral characteristics of amyloid β in the retina^16,17^. Multispectral autofluorescence (MAF) technology advances this field by enabling the high-precision, simultaneous resolution of multiple physiological parameters^18^. Compared with single-spectrum imaging, MAF offers distinct advantages in signal dimensionality, tissue specificity, and clinical translational potential^19^, forming a critical technical foundation for label-free pathological diagnosis.

The integration of digital pathology and deep learning, together with the broader integration of artificial intelligence (AI) and pathology, has not only catalyzed the advent of virtual staining but also profoundly transformed traditional diagnostic workflows^20–24^. As a pivotal innovation in this interdisciplinary space, virtual staining is a novel computational methodology rooted in image-to-image transformation frameworks and leverages deep learning algorithms to mimic the efficacy of conventional chemical staining. It directly converts rapidly acquired label-free images, most notably autofluorescence images that contain rich, complex metabolic and pathological information, into diagnostically actionable, H&E-equivalent images that are readily interpretable by pathologists^25,26^. First, it advances pathology practices. This technology enables the end-to-end generation of diagnostic outputs and a seamless transition from “label-free imaging to diagnostic-grade stained images”. Beyond this core function, it preserves sample integrity, enhances staining efficiency, and retains critical biological samples for downstream applications^27^. Notably, virtual staining harbors considerable clinical potential, and its utility is particularly pronounced in surgical settings. Here, integrating portable MAF imaging devices with edge computing enables the real-time intraoperative generation of virtual hematoxylin and eosin (vH&E) images. This capability provides surgeons with critical pathological guidance while significantly reducing the time burden associated with intraoperative pathological assessment. Building on this unique advantage, intraoperative rapid pathological diagnosis has thus emerged as a key future direction for advancing this domain.

The rising global incidence of breast cancer and its increasing onset in younger populations underscore the urgent need for more accurate preclinical models to advance individualized therapeutic strategies^28^. Tumor-bearing mouse models are pivotal for mechanistic research, as they highly recapitulate tumor biological characteristics, provide a controlled microenvironment for tumor growth, support quantitative assessment of targeted interventions, and enable standardized pharmacodynamic analyses^29,30^. Recent innovations in organoid modeling have further expanded the research landscape. Using pluripotent stem cells or primary tissue samples, organoid technology constructs in vitro models that faithfully replicate native tissue 3D architecture and functional properties, serving as a high-fidelity platform for designing individualized regimens^31–36^.

To enhance cross-model translational efficiency, a framework for deep integration with virtual staining technology has been proposed. Virtual staining enables quantitative analysis of diagnostically equivalent images, supports downstream analyses via non-destructive sample preservation, and maintains the integrity of unstained tissues for molecular assays, thus preserving precious biological information. As the first framework to systematically integrate virtual staining technology across diverse breast cancer research models, including tumor-bearing mice, organoids, and clinical samples, this approach establishes a seamless workflow spanning label-free imaging, diagnostic-grade image generation, and multimodal data analysis. This workflow overcomes the sample loss and technical barriers associated with traditional staining methods. Ultimately, it provides a retrospective and scalable precision medicine research platform, bridging the gap between preclinical discovery and clinical application.

The main contributions of this study are: (1) we developed a novel virtual staining algorithm based on MAF imaging and an optimized CycleGAN architecture. This framework enables the end-to-end transformation of unlabeled tissue sections into diagnostic-grade H&E images without the need for paired training data, while offering a notable clinical advantage. It accelerates workflows by generating vH&E images within minutes, which markedly enhances the efficiency of pathological image acquisition and exhibits substantial potential for the real-time assessment of intraoperative tumor margins. (2) We pioneered the systematic compilation of an MAF dataset encompassing human breast cancer tissues, breast cancer-lung metastasis organoid co-cultures, and breast cancer-bearing murine models; this dataset represents the first multispectral database of its kind tailored to multi-scale breast cancer biological systems. Additionally, it provides a foundational resource for training robust AI models dedicated to multi-model pathological analysis, thereby accelerating the development of next-generation diagnostic tools. (3) We engineered a cross-model adaptive framework that integrates the virtual staining algorithm with the multi-model MAF database. This integration facilitates the robust extraction of pathology-invariant features through advanced cross-scale feature disentanglement. By leveraging the database’s rich spectral-pathological correlations, the framework enables high-fidelity virtual staining across diverse biological systems. A key advantage of this framework lies in its ability to facilitate the “single-sample, multi-omics” paradigm. Specifically, preserving tissue biomolecular integrity ensures the same unstained sample used for virtual staining remains suitable for subsequent single-nucleus RNA sequencing or spatial transcriptomics.

## Results

In this study, a comprehensive and systematic evaluation was conducted on the MAF-based virtual staining system. Results indicate that this system not only generates pathological images with high consistency relative to those obtained via conventional chemical staining but also exhibits robust cross-model transferability across diverse biological models. This finding validates the system’s broad applicability in both preclinical research and clinical practice settings.

### Quantification of photonic dose and impact on molecular integrity

To objectively address the potential for photodamage during MAF imaging, we quantified the photonic exposure, particularly for the DAPI channel (excitation: 377/50 nm), which utilizes light in the near-UV spectrum. The irradiance at the sample plane was calibrated to 8 mW/cm², with an exposure time of 5 s per field of view. The total fluence (energy dose per unit area) was calculated as follows: Fluence = Irradiance × Time=8 mW/cm² × 5 s = 40 mJ/cm².This dose is considered minimal for fixed tissues. For context, it is orders of magnitude lower than the fluence thresholds known to induce significant nucleic acid crosslinking or photolysis. Therefore, while any optical imaging involves energy deposition, the photonic burden of our MAF protocol is negligible. This ensures the preservation of molecular integrity for downstream analyses.

### Visual comparison of virtual stained images

**Fig. 1:**
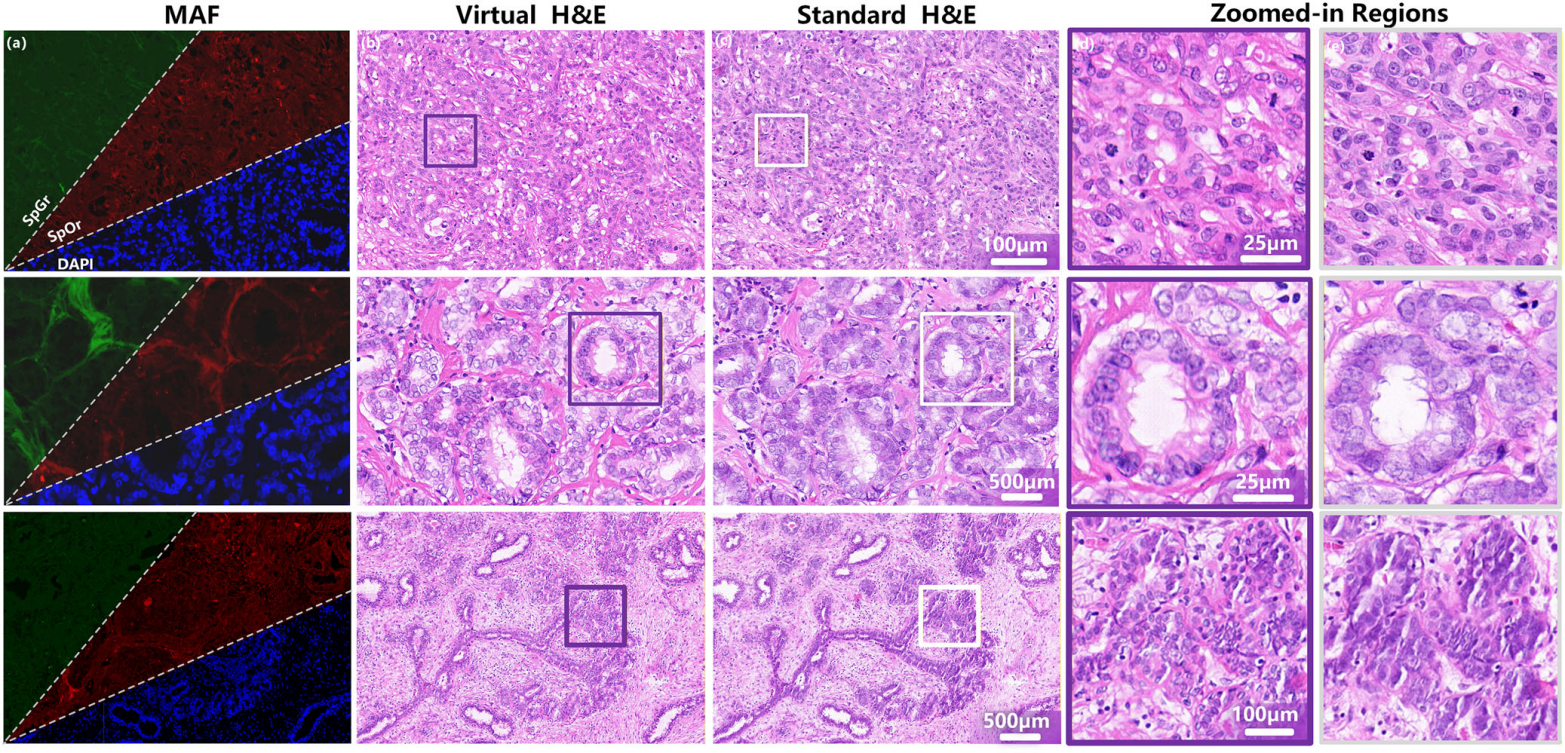
Visual comparison of label-free MAF, vH&E, standard H&E, and their zoomed-in regions across diverse breast cancer histological contexts, demonstrating morphological fidelity of vH&E to standard H&E at multiple scales. **a** Label-free MAF images composed of three channels: SpGr, SpOr, and DAPI. These channels provide intrinsic tissue molecular information, serving as the input for the virtual staining model. **b** Images generated by deep learning from (**a**) inputs, designed to mimic the staining pattern of standard H&E.**c** Conventional chemically stained histology images, used as the gold standard for morphological validation. **d**, **e** High-magnification views of the boxed areas—purple boxes in (**b**) and white boxes in (**c**), respectively—enabling detailed comparison of fine structural features between vH&E and standard H&E.

First, a direct visual comparison was performed between vH&E images generated by the model and real H&E images derived from the same tissue section, as presented in Fig. 1. This comparison revealed that the model exhibited superior performance in preserving key diagnostic morphological features. Specifically, well-differentiated ductal glands growing within sclerotic fibrous stroma were accurately recapitulated, and the ductal structure, composed of ductal epithelial cells and myoepithelial cells, was clearly discernible. Additionally, the presence of “popcorn-like nuclei,” vacuolated nuclei, and mitotic figures in the vH&E images confirmed that the virtual staining approach effectively characterizes markers of nuclear atypia and cellular proliferation, which are critical for breast cancer pathological diagnosis.

**Fig. 2:**
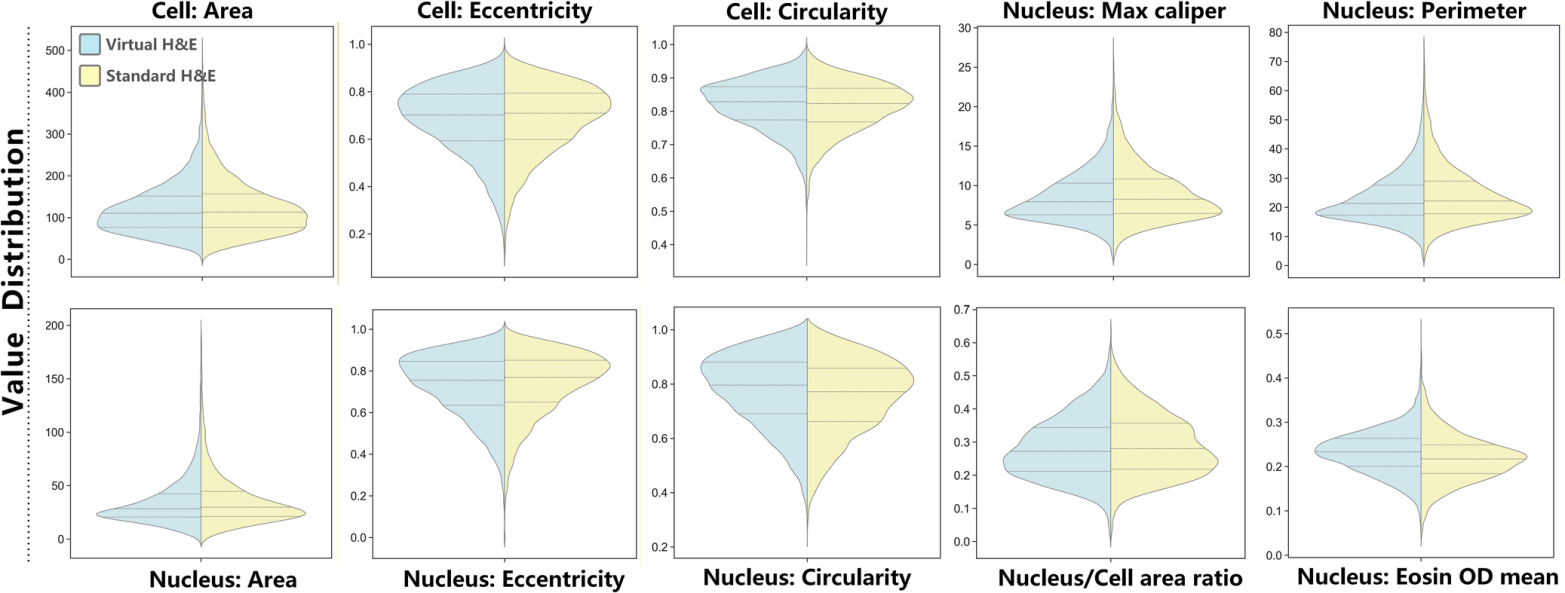
Quantitative morphological comparison of vH&E and standard H&E staining in breast cancer histopathology, visualized via violin plots of 10 key morphological parameters. Each panel illustrates the distribution of values for vH&E (light blue) and standard H&E (yellow) across. Cellular features: Cell Area, Cell Eccentricity, and Cell Circularity. Nuclear features: Nucleus Max Caliper, Nucleus Perimeter, Nucleus Area, Nucleus Eccentricity, Nucleus Circularity, Nucleus/Cell Area Ratio, and Nucleus Eosin Optical Density (OD) mean.

### Quantitative assessment of virtual staining

To address the limitations of subjective visual assessment and objectively validate the reconstruction accuracy of diagnostically critical features, we performed precise semantic segmentation of cells and nuclei, followed by the extraction of ten key morphometric and optical density (OD) metrics. This enabled systematic quantification of the fidelity with which vH&E images recapitulate histological features of standard H&E staining.

As illustrated in Fig. 2, vH&E (blue) exhibited remarkable consistency with standard H&E (orange) in the distribution of all evaluated parameters. Specifically, for core cellular features including cell area and nuclear area, the violin plots demonstrated substantial distribution overlap with congruent peak shapes—quantified by high Jaccard overlap coefficients (0.89 ± 0.03 and 0.91 ± 0.02), low Jensen–Shannon (JS) divergence (<0.12 for both), non-significant Kolmogorov–Smirnov (KS) test results (*p* = 0.78 and *p* = 0.83), and negligible effect sizes (Cohen’s *d* = 0.08 and 0.06). These statistical metrics confirm that cell and nuclear area distributions from vH&E are statistically indistinguishable from standard H&E. Consistent findings were observed for nuclear circularity (overlap coefficient = 0.87 ± 0.04, KS *p* = 0.69, Cohen’s *d* = 0.09) and eccentricity (overlap coefficient = 0.88 ± 0.03, KS *p* = 0.72, Cohen’s *d* = 0.10), validating that vH&E faithfully preserves cell-scale morphological integrity.

For diagnostically actionable features, vH&E showed high concordance with standard H&E in nuclear perimeter (overlap coefficient = 0.90 ± 0.03, KS *p* = 0.75), maximum nuclear caliper (overlap coefficient = 0.88 ± 0.04, KS *p* = 0.67), and nuclear-to-cytoplasmic (N/C) area ratio (overlap coefficient = 0.92 ± 0.02, KS *p* = 0.81). Notably, the precise matching of N/C ratio—a core parameter for Nottingham modified Scarff-Bloom-Richardson (SBR) grading—demonstrated that vH&E can reliably identify clusters of abnormally enlarged nuclei and distinguish between low- and high-grade lesions. This is clinically critical, as SBR grading directly informs prognostic stratification and treatment decision-making for breast cancer patients.

Comprehensive statistical analysis further confirmed that all morphometric features (cell area, nuclear area, nuclear perimeter, maximum nuclear caliper, circularity, eccentricity, N/C ratio) were statistically congruent with standard H&E (KS *p* > 0.05, Cohen’s *d* < 0.1). In contrast, staining intensity-related metrics—specifically nuclear Hematoxylin OD mean and cytoplasmic Eosin OD mean—exhibited statistically significant differences (KS p < 0.01) with moderate effect sizes (Cohen’s d = 0.53 and 0.59). Nevertheless, their distribution shapes remained highly similar (JS divergence=0.25 and 0.28), and the consistent offset in absolute OD values reflects a calibration challenge rather than a fundamental failure of morphological reconstruction. This discrepancy may stem from inherent batch-to-batch variability in chemical staining protocols or the complex non-linear relationship between endogenous tissue fluorophores and dye absorption—limitations inherent to the current CycleGAN architecture.

Collectively, these findings validate that vH&E accurately recapitulates morphometric features critical for breast cancer diagnosis and SBR grading, with statistical equivalence to standard H&E for all structural metrics. The observed limitations in staining intensity are addressable through emerging modeling advancements, reinforcing the potential of vH&E as a reliable, label-free alternative to conventional chemical staining in clinical and preclinical breast cancer research.

### Clinical validation of virtual staining

**Fig. 3:**
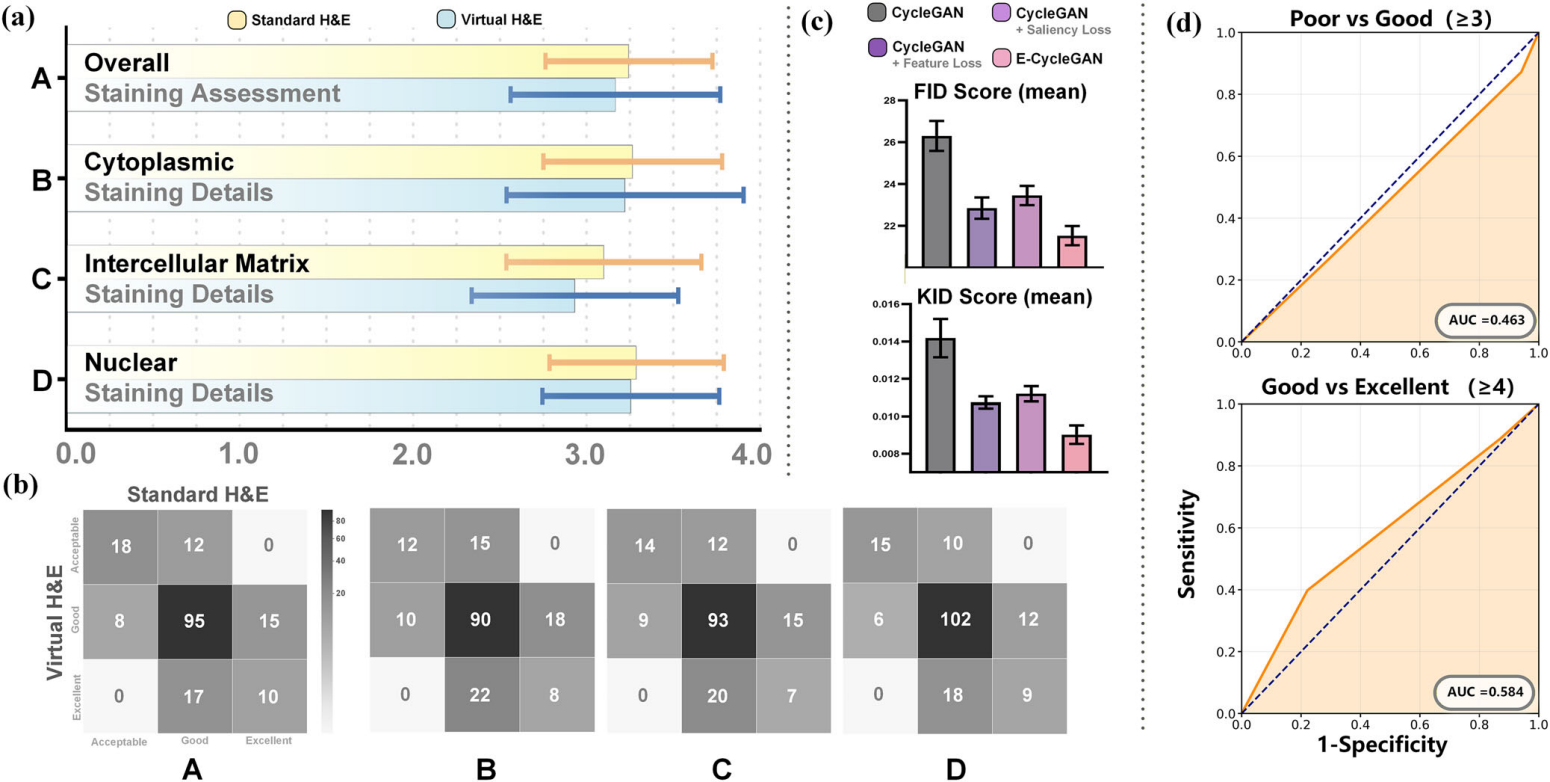
Multidimensional evaluation of vH&E staining quality, algorithmic performance, and clinical indistinguishability. **a** Pathologist-assessed scores for four histologic features—overall staining assessment (A), cytoplasmic staining details (B), intercellular matrix staining details (C), and nuclear staining details (D)—comparing standard H&E (yellow) and vH&E (light blue). Error bars reflect variability across five board-certified pathologists, demonstrating that vH&E scores are statistically congruent with standard H&E across all evaluated features. **b** Confusion matrices for the same four features (A–D), with rows denoting vH&E scores and columns denoting standard H&E scores. Darker cells indicate higher counts of matching scores, quantifying the high concordance between vH&E and standard H&E in clinical grading. **c** Fréchet Inception Distance (FID) and Kernel Inception Distance (KID) scores for different CycleGAN variants: baseline CycleGAN, CycleGAN + Saliency Loss, CycleGAN + Global Feature Loss, and our optimized E-CycleGAN. Lower scores indicate closer similarity to real (standard H&E) images, confirming that E-CycleGAN outperforms other variants in preserving morphological fidelity. **d** Receiver operating characteristic (ROC) curves for distinguishing “vH&E vs. standard H&E” in two grading scenarios: Poor vs Good (score ≥ 3, top panel) and Good vs Excellent (score ≥ 4, bottom panel). Area under the curve (AUC) values near 0.5 (dashed line = random chance) indicate that vH&E is clinically indistinguishable from standard H&E, validating its diagnostic equivalence.

To assess the clinical applicability of the proposed virtual staining framework, a quantitative blinded study was conducted. Eighteen pairs of vH&E and conventional H&E staining images were randomly selected from the dataset. Uniform preprocessing was applied to the images, including random rotation of ±90°, horizontal/vertical flipping, and sequence rearrangement, after which the images were combined to form a blinded review dataset. Five experts in breast pathology diagnosis were invited to conduct a double-blind evaluation. Each image was independently scored based on four preset histologic features, namely nuclear staining details, cytoplasmic staining details, intercellular matrix staining details, and overall staining assessment, using a 4-point scale (1 = unacceptable, 2 = acceptable, 3 = good, 4 = excellent). An arbitration review was initiated if score differences exceeded 2 grades. Statistical analysis using Wilcoxon signed-rank tests revealed no statistically significant differences between vH&E and conventional H&E images across all evaluated features. For nuclear staining details, the test yielded a rank sum statistic (*W*) of 289.5 with a two-tailed *p* value of 0.64, indicating that the distribution of scores between vH&E and standard H&E was nearly identical. Similarly, cytoplasmic staining details (*W* = 371.0, *p* = 0.556), intercellular matrix staining details (*W* = 448.0, *p* = 0.77), and overall staining assessment (*W* = 290.5, *p* = 0.312) all showed *p* values > 0.05, confirming that vH&E staining performance was statistically equivalent to standard H&E across all histologic dimensions. These results not only demonstrate the absence of significant scoring discrepancies but also reflect the high concordance in rank distributions, reinforcing that vH&E can reliably recapitulate the diagnostic morphological features assessed by pathologists. Quantitative results from the confusion matrix further validated the performance of vH&E staining. In terms of overall staining performance, 82.2% of vH&E images achieved scores no lower than those of standard H&E images; for nuclear staining details, this proportion reached 83.3%. Across all scoring dimensions, nearly 80% of vH&E images demonstrated performance equivalent to or better than standard H&E staining. To further quantify the ability to distinguish between vH&E and standard H&E staining, we performed receiver operating characteristic (ROC) analysis. We framed this as a binary classification task: distinguishing “vH&E” from “standard H&E” based on the four histologic feature scores. Two analytical scenarios were defined: (1) “Poor vs Good” (with “Good” defined as a composite score ≥3) and (2) “Good vs Excellent” (with “Excellent” defined as a composite score ≥4). As presented in Fig. 3d, the ROC curves for vH&E were compared against a random classifier. For “Poor vs Good” classification, vH&E achieved an area under the curve (AUC) of 0.463, with a sensitivity of 0.872 and specificity of 0.059. For “Good vs Excellent”, the AUC was 0.584, with a sensitivity of 0.398 and specificity of 0.779. These AUC values are close to 0.5, indicating that the distinction between vH&E and standard H&E is statistically indistinguishable from random chance. This outcome demonstrates that vH&E staining is so similar to standard H&E that it cannot be reliably distinguished, confirming its excellent performance and clinical equivalence. These findings fully indicate that vH&E staining is equivalent to standard H&E staining in its core capacity to visualize cellular microstructures and can fully meet the basic clinical requirements for pathological image review. Detailed information can be found in the supplementary materials.

### Comparative experiments

**Fig. 4:**
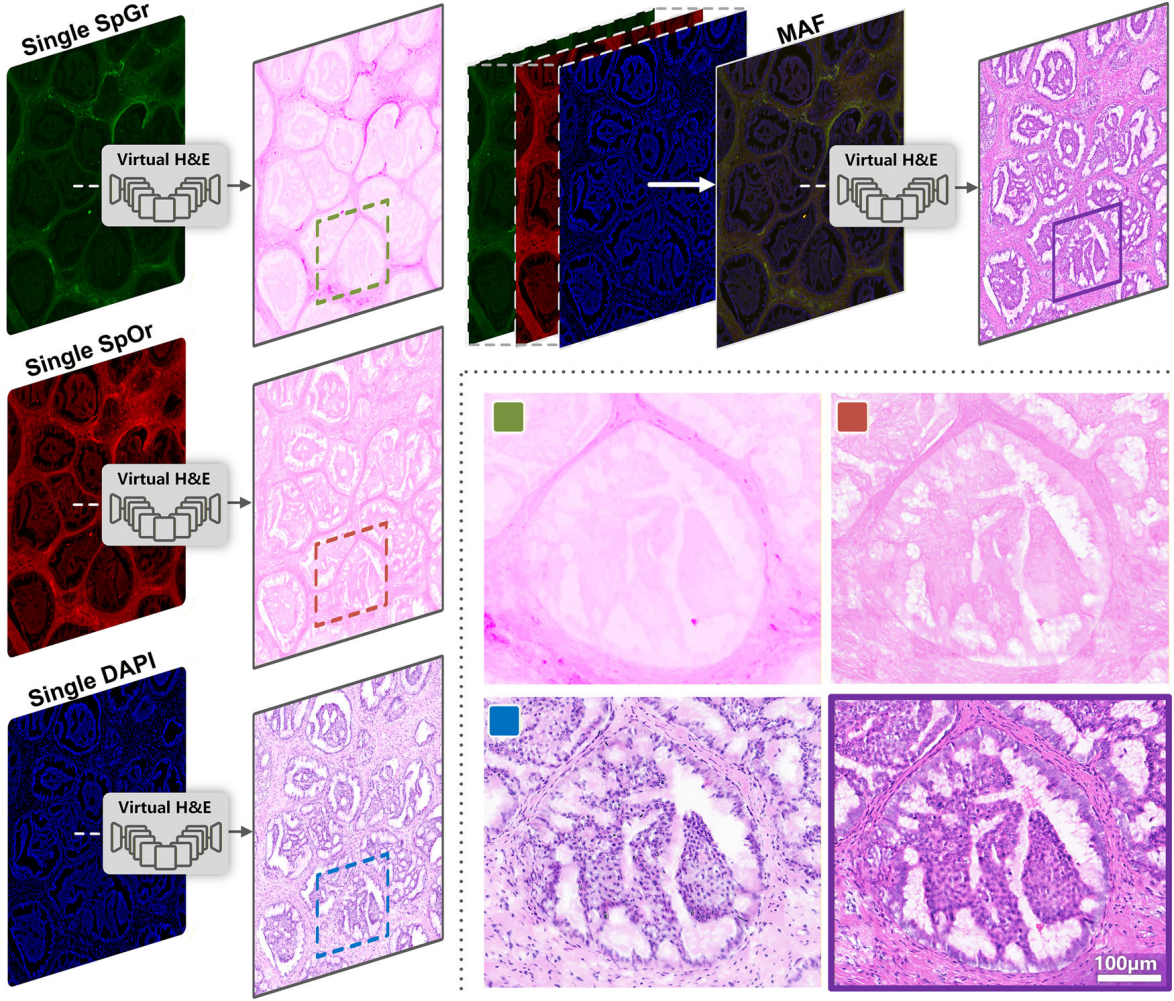
Comparative analysis of single-channel vs. multispectral virtual H&E staining, validating the necessity of multispectral fusion for morphological fidelity in breast cancer histopathology. Left panels: Virtual H&E staining results derived from single-channel inputs: Single SpGr (green channel), Single SpOr (red channel), and Single DAPI (blue channel). Each single-channel input is processed through the virtual staining pipeline, with the resulting vH&E images (right of each single-channel panel) exhibiting blurred tissue boundaries, color distortion, and loss of fine structural details (highlighted by dashed boxes). Right upper panels: MAF imaging, integrating multiple spectral channels (illustrated by overlapping green, red, and blue channels), enables robust virtual H&E staining. The resulting vH&E image (far right) demonstrates sharp tissue architecture, accurate nuclear/cytoplasmic contrast, and preservation of diagnostic features (marked by the purple box). Bottom panels: High-magnification comparison of vH&E from single-channel (left, green/red/blue boxes) vs. MAF-based multispectral (right, corresponding colored boxes) inputs. These panels emphasize that multispectral fusion resolves the color distortion and detail loss inherent to single-channel approaches, delivering vH&E images with fidelity to standard H&E staining.

We further compared the improved algorithm with traditional algorithms. As shown in Fig. 3c and detailed in Table 1, quantitatively, objective metrics confirmed clear performance improvements: Fréchet Inception Distance (FID) decreased by 18.15%, which reduces the feature distribution distance between generated images and real pathological sections and verifies that spatial-spectral joint modeling achieves a qualitative enhancement in color rendering and structural reproduction. Additionally, Kernel Inception Distance (KID) decreased by 36.39%, which reflects a reduction in Maximum Mean Difference (MMD) of generated images in the nuclear-related feature space and validates that the algorithm improves the reconstruction accuracy of microstructures such as nuclear morphology and chromatin distribution. To systematically validate the necessity of multispectral information fusion, we compared the proposed model with a baseline model that uses only a single fluorescence channel as input. As shown in Fig. 4, images generated via the single-spectrum method exhibited severe color distortion and detail loss. Specifically, target cellular components and mesenchymal staining lacked clear differentiation; key features, including cell membranes, cytoplasm, and nuclei, were not adequately visualized; lymphocyte clusters in the mesenchyme and in situ cancer foci with ductal hyperplasia appeared structurally indistinct due to insufficient spectral contrast; and blurred nucleoplasmic boundaries rendered pathological diagnostic features uninterpretable. In contrast, images from our multispectral model demonstrated a qualitative leap in color accuracy, contrast, and structural detail preservation, which highlights the core advantages of multispectral imaging for label-free pathological diagnosis.

**Table 1:** Comparison of different model variants against real H&E images using FID and KID metrics

Model variant	FID score	KID score
E-CycleGAN (Ours)	21.537 ± 0.522	0.0090 ± 0.0005
CycleGAN + Feature Loss	22.835 ± 0.631	0.0107 ± 0.0005
CycleGAN + Saliency Loss	23.456 ± 0.750	0.0114 ± 0.0004
CycleGAN (Baseline)	26.308 ± 0.673	0.0142 ± 0.0009

Overall, multispectral information fusion overcomes the inherent limitations of single-spectral channels, enabling breakthroughs in cellular substructure visualization and diagnostic information preservation. The improved algorithm meets the quality standards for clinically usable pathological images, thereby providing a novel technical pathway for label-free pathological diagnosis.

### Application and validation of virtual staining in multi-model systems

**Fig. 5:**
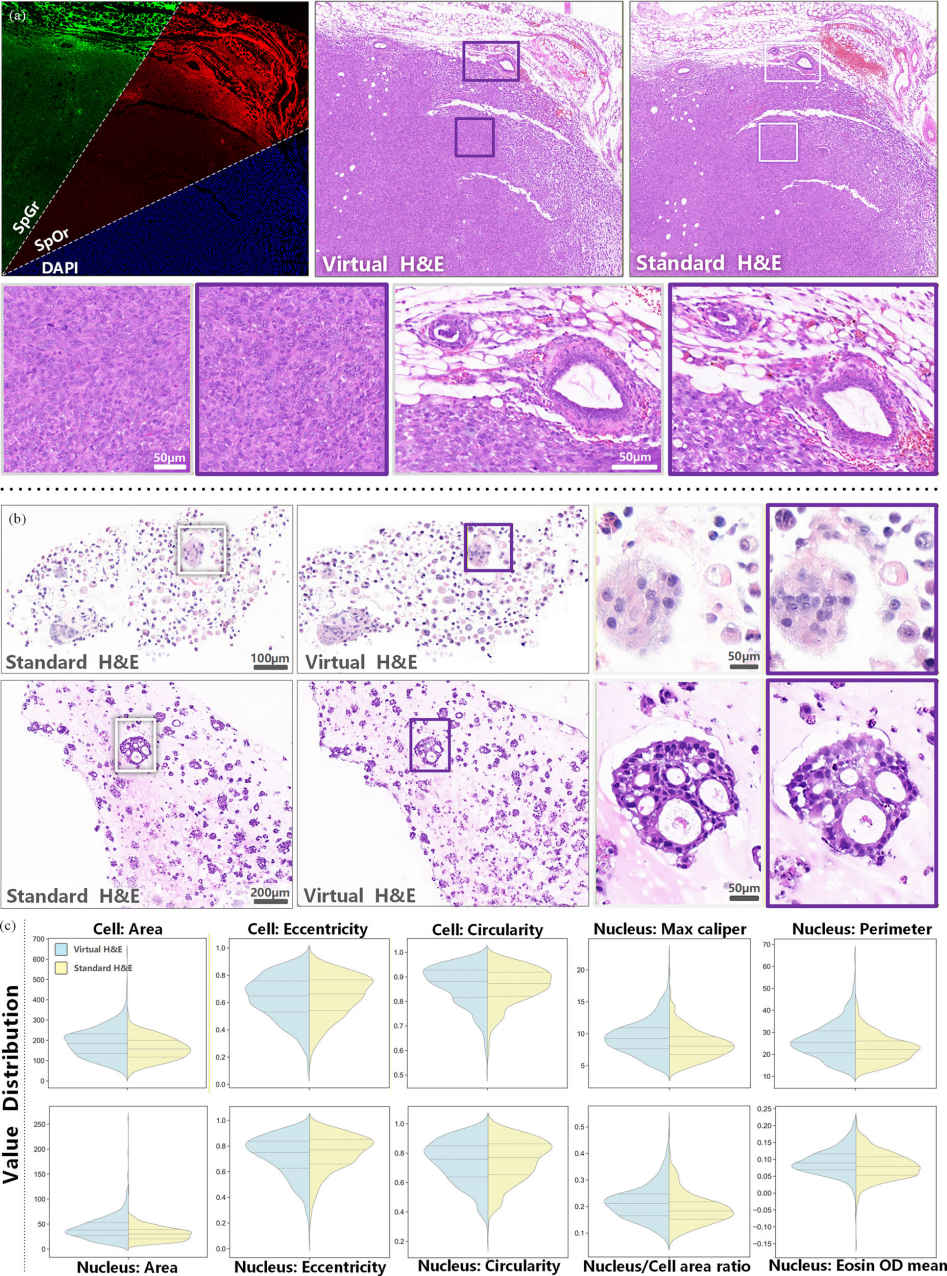
Application of vH&E staining across multi-model breast cancer systems, demonstrating morphological and quantitative fidelity in preclinical research. **a** Three-channel multispectral fluorescence in a mouse orthotopic breast cancer model. Top-left panel: Label-free multispectral fluorescence image composed of three channels—SpGr, SpOr, and DAPI—from a mouse orthotopic breast cancer model. Adjacent panels compare vH&E (middle) and standard H&E (right) staining of the same tissue. Below these, zoomed-in regions highlight vH&E’s capacity to recapitulate tumor-stroma architecture, cellular density, and nuclear-cytoplasmic contrast in murine tumor tissue, matching the detail of standard H&E. **b** Breast cancer-lung organoid co-culture system. Upper and lower subpanels illustrate standard H&E and vH&E staining of breast cancer-lung organoid co-cultures. vH&E faithfully reproduces key features of these 3D systems, including organoid morphology, cellular clustering, and fine structural details as observed in standard H&E. Zoomed-in regions illustrate the side-by-side detail comparison between vH&E and standard H&E, highlighting how vH&E precisely captures diagnostic features with fidelity to standard H&E. **c** Quantitative morphological comparison in organoid co-culture models. Violin plots visualize the distribution of 10 key morphometric parameters for vH&E (light blue) and standard H&E (yellow) in breast cancer-lung organoid co-cultures.

To demonstrate the cross-species applicability of our algorithm, we initially conducted experiments across multiple distinct breast cancer models, including mouse orthotopic breast cancer models, using MAF and H&E images for model training and validation. Results demonstrated that the framework successfully learned and adapted to cross-species pathological features, enabling robust feature transfer. The generated vH&E images accurately reproduced critical pathological hallmarks. First, they captured tumor heterogeneity and necrotic regions, where epithelioid tumor cells appeared in dense solid clusters with moderate size variation, and local cells exhibited ovoid or short spindle shapes with suspected nesting arrangements. Second, they clearly visualized adipose tissue infiltration at tumor margins and suspected intravascular thrombi. Furthermore, they preserved high-fidelity nuclear characteristics, including prominent vacuolated nuclei and large eosinophilic nucleoli. As shown in Fig. 5, vH&E images achieved high consistency with real H&E images, confirming the framework’s strong cross-species applicability that is not limited to samples of specific origin.

Similarly, we validated the feasibility of this method in a breast cancer-lung organoid co-culture model. As shown in Fig. 5, post-training virtual staining enabled non-destructive 3D reconstruction of the co-cultures, clearly visualizing the growth of cancer cell clusters and their interactions with the surrounding matrix. The images revealed discontinuous monolayer epithelia in localized regions, homogeneous pink staining bands in lung organoids during early branching morphogenesis, and scattered breast cancer cells at the periphery of organoids. For breast cancer organoids constructed using the same method, the virtual staining approach successfully preserved key pathological features, including high cellular and nuclear pleomorphism, as well as primary breast cancer cells with nested hyperplasia.

These results confirm that our MAF imaging and virtual staining technique serves as an innovative morphological assessment tool for complex organoids. Its non-destructive and rapid nature supports in vitro drug sensitivity screening and tumor invasion research, while preserving specimens for downstream molecular analyses. Our workflow’s tissue-preserving design enables a sequential analysis paradigm: MAF imaging and virtual staining-based diagnosis, and the identical tissue section can be subjected to single-nucleus RNA sequencing or spatial transcriptomics. By generating diagnostically equivalent H&E-like images without chemical staining or tissue modification, our approach prioritizes maximizing tissue preservation to unlock its full utility. With the advancement of multispectral technology, this approach may enable dynamic in vitro organoid monitoring and multi-omics integration, laying a foundation for correlating morphological changes with molecular signatures.

## Discussion

This study presents a transformative label-free vH&E staining framework that integrates MAF imaging with unsupervised AI trained on unpaired images. Validated across clinical breast cancer sections, murine orthotopic tumor models, and organoid co-culture systems, this framework addresses long-standing limitations of traditional H&E staining, including cumbersome workflows and irreversible tissue alteration, thereby representing a paradigm shift in clinical pathology with profound implications for precision oncology and translational research^37^.

Its unsupervised learning mechanism, trained on unpaired data, eliminates reliance on large-scale paired datasets, which have long been a major bottleneck in digital pathology; this is achieved by learning intrinsic mappings between spectral features and histological structures^38^. This adaptability to real-world clinical variability lowers deployment barriers while delivering three core clinical benefits. First, it advances green pathology practices. Our virtual staining technique replaces the carcinogenic reagents used in the conventional H&E staining protocol. Moreover, as quantitatively demonstrated, the optical imaging process itself imposes a minimal photonic burden, significantly preserving nucleic acid integrity. This reduces occupational health risks for laboratory personnel associated with the staining process and minimizes the environmental footprint of chemical staining workflows. This alignment with sustainable healthcare goals marks an important step toward eco-friendly pathological workflows. Second, it enhances diagnostic efficiency: staining workflows are compressed to minutes, an improvement critical for time-sensitive scenarios such as intraoperative frozen section analysis. In these contexts, rapid and accurate results directly guide surgical decision-making, which can minimize unnecessary invasive procedures and improve patient outcomes. Both qualitative assessments by experienced pathologists and quantitative image analyses confirm that virtual staining images match traditional H&E staining in key diagnostic indicators, including nuclear details, chromatin distribution, and tissue architecture, ensuring the virtual images meet clinical diagnostic standards and remain reliable in high-stakes decision-making. Third, the framework’s virtual staining technology also supports rapid pathological diagnosis of organoids. This rapidity addresses a key bottleneck in organoid research: traditional staining workflows, with their long turnaround times, often delay the assessment of organoid morphology and pathological features. By contrast, virtual staining delivers diagnostic-grade images in minutes, allowing researchers to quickly evaluate organoid integrity, identify abnormal phenotypes, and monitor dynamic changes in response to experimental interventions (e.g., drug treatments or microenvironmental perturbations).

Comparative experiments confirm that multispectral integration outperforms single-spectrum approaches. Specifically, single-spectral input, due to its lack of complementary endogenous fluorophore information, leads to blurred cytoplasmic boundaries, color distortion, and obscured nuclear assessment. In contrast, the spectral unmixing capability of the MAF system captures rich tissue metabolic and structural details, enabling end-to-end optical mapping of H&E staining patterns, including the characteristic hematoxylin-nuclear blue and eosin-cytoplasmic red^39,40^.

Across multimodal breast cancer models, the virtual staining system exhibits robust applicability and significant translational value. In 4T1 murine models, it accurately restores key pathological features, including tumor heterogeneity, necrotic foci, and immune infiltration, supporting reliable preclinical assessment of tumor biology^41^. In breast cancer-lung organoid co-cultures, the system further delivers dual advantages: it enables sample multiplexing, a capability with particular utility for advancing organoid research. By preserving tissue integrity for post-imaging downstream analyses (e.g., single-cell sequencing, spatial transcriptomics, and proteomics), virtual staining maintains high specimen quality. This attribute not only lays a stronger foundation for personalized treatment strategies but is also especially critical for rare or small-volume samples, such as core needle biopsies and primary cell-derived organoids. Additionally, the system facilitates “single-sample, multi-insight” characterization by linking morphological phenotypes, visualized via virtual staining and encompassing both clinical tissue and organoid features, with molecular signatures. This allows researchers to deepen their understanding of breast cancer pathogenesis, tumor microenvironment dynamics, and therapeutic resistance mechanisms. This integration bridges the long-standing gap between histology and molecular biology, while the system’s accelerated organoid diagnostic workflow further expedites translational research, moving preclinical findings closer to clinical application.

During the image review process, pathologists observed that vH&E staining exhibited excessively high uniformity in cytoplasmic staining in some samples, which did not fully align with the inherent morphological variability of native cytoplasm. A related limitation is the limited sensitivity of MAF to cytoplasmic components, which impedes the accurate restoration of cytoplasmic morphology. Specifically, MAF shows insufficient responsiveness to cytoplasmic endogenous fluorophores, further hindering the differentiation of subcellular structures such as the Golgi apparatus and endoplasmic reticulum, components critical for subtle pathological evaluations. To address this, future iterations of the framework could integrate multiphoton microscopy with second harmonic generation to enhance collagen fiber contrast, or incorporate stimulated Raman scattering to complement chemical specificity for lipids and proteins^42^. These modifications would enrich cytoplasmic detail and improve subcellular visualization. Additionally, the current reliance on single-center data restricts the framework’s generalizability. Incorporating multi-center, multi-device MAF datasets will therefore be essential to validate the system’s consistency across diverse clinical settings, supporting regulatory approval and facilitating widespread clinical adoption. Long-term advancements could involve integrating MAF live scanning devices with edge computing to enable real-time, in situ virtual staining directly in the surgical field^43,44^. This integrated technology would deliver immediate, high-fidelity histological insights to pathologists and surgeons during procedures, including intraoperative margin assessment and lymph node dissection guidance, addressing critical unmet needs for rapid, actionable pathological information in the operating room^45^.

By facilitating data-driven decision-making during surgery, such capabilities would not only revolutionize personalized surgical planning but also have the potential to optimize treatment precision and ultimately improve long-term patient outcomes.

## Methods

### Image data acquisition

The MAF images of unstained tissue sections were captured using a fluorescence slide scanner (Pannoramic MIDI, 3DHISTECH) equipped with a Plan-Apochromat 20× objective lens. Three fluorescent filter sets were employed: Dapi-5060-C-ZERO (EX 377/50 nm, EM 477/60 nm), SpGreen-ZERO (EX 494/20 nm, EM 527/20 nm), and SpOr_Zero (EX 543/22 nm, EM 586/20 nm). Each MAF image was acquired with a scientific complementary metal-oxide-semiconductor image sensor integrated into a Point Grey GS3-U3-51S5M-C camera. The image acquisition process was controlled by scanner software (version 1.23.1.71684), with flat field correction enabled to ensure image uniformity.

### Multispectral fluorescence imaging of clinical specimens

**Fig. 6:**
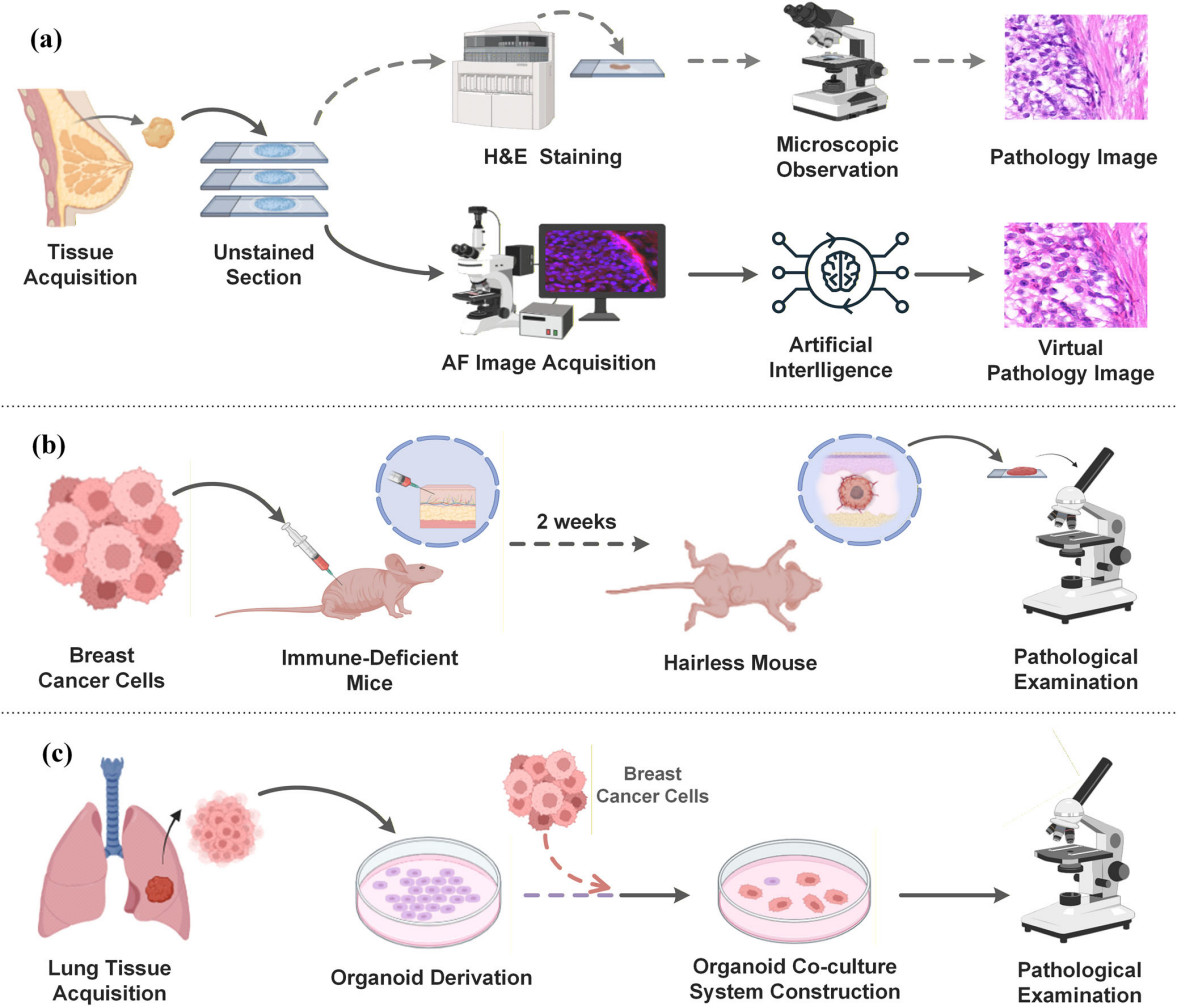
Technical workflow diagrams illustrating image acquisition pipelines and experimental model construction for breast cancer histopathological analysis. **a** Clinical section workflow: depicts the parallel pipelines for generating standard H&E and virtual pathology images from clinical breast tissue sections. **b** Mouse orthotopic breast cancer model construction and image acquisition workflow: outlines the establishment and histopathological analysis of a mouse orthotopic breast cancer model. **c** Breast cancer-lung organoid co-culture model construction and image acquisition workflow: outlines the creation of a 3D co-culture system to mimic breast cancer-lung interactions and subsequent histopathological image acquisition. (Created with BioRender.com. Sun, J. (2025). https://BioRender.com/d33j2op).

The study was conducted in accordance with the Declaration of Helsinki. It was approved by the Ethics Committee of Chinese PLA General Hospital with protocol codes S2024-711-01 (date of approval: November 28, 2024) and S2025-419-01 (date of approval: May 29, 2025) for studies involving human participants. Written informed consent was obtained from all participants or their legal representatives prior to sample collection. Freshly isolated breast cancer tissue specimens were fixed in 4% paraformaldehyde for 24 h to preserve tissue morphology. Following fixation, specimens were processed using a standard workflow. This involved dehydration with a graded ethanol series to ensure complete water removal, followed by xylene-based transparency treatment to eliminate residual ethanol. Transparent specimens were then embedded in paraffin wax using a tissue embedding machine to form paraffin blocks, which were subsequently sectioned into 4μm serial sections using a Leica RM2016 microtome. Each section was floated on 40 °C warm water for natural flattening to avoid artificial folds and retrieved with anti-dehiscence pre-treated glass slides. Slides with adherent sections were baked at 60 °C in a DGX-9003B oven to enhance tissue-slide adhesion, after which they were allowed to cool naturally to room temperature. Cooled slides were scanned using a Pannoramic MIDI full-slide scanner (3DHISTECH, Hungary) to acquire MAF signals from unstained sections. Detailed acquisition parameters are provided in Table 2. To ensure spatial and morphological consistency between MAF images and H&E images, sections were deparaffinized with xylene and rehydrated via a reverse graded ethanol series after MAF signal acquisition. Residual ethanol was removed by rinsing sections in distilled water, and the rehydrated sections were stained using a standard H&E protocol to generate ground-truth histological images. Relevant experimental procedures are shown in Fig. 6.

**Table 2:** Autofluorescence imaging parameters for multi-channel spectral analysis

Fluorescence channel	Excitation light/Excitation bandwidth (nm)	Emitted light/Emission bandwidth (nm)	Dichroic mirror (nm)
DAPI	377/50	477/60	409
SpGr	494/20	527/20	506
SpOr	543/22	586/20	562

### Multispectral fluorescence data acquisition for mouse breast cancer

The study involving animals was approved by the Ethics Committee of Beijing Viewsolid Biotechnology Co. LTD (protocol code: VS2126A00750, date of approval: February 1, 2025), and all procedures were performed in compliance with institutional animal care and use guidelines to minimize animal suffering. Female BALB/c-nu mice (6–8 weeks old) were acclimatized for 1 week in a barrier environment under controlled conditions, including a temperature of 22 ± 2 °C, a relative humidity of 50 ± 10%, and a 12 h light/dark cycle. During acclimatization, mice had free access to sterilized food and water to minimize environmental stress before experiments. Under aseptic conditions, 4T1-luc breast cancer cells were resuspended in phosphate-buffered saline (PBS) to a final concentration of 1 × 10⁷ cells/mL. A total of 100 μL of the cell suspension was slowly injected subcutaneously into the right fourth mammary fat pad of each mouse using a 1 mL sterile syringe with a 27 G needle, establishing an orthotopic xenograft tumor model. After tumor cell inoculation, tumor growth was monitored every 3 days. The tumor’s long diameter (a) and short diameter (b) were measured using digital vernier calipers (Mitutoyo), and tumor volume was calculated with the standard formula: *V* = (*π* × *a* × *b*²)/6. When tumor volumes reached ≥400 mm³, mice were euthanized in accordance with the Animal Ethics Guidelines approved by the Institutional Animal Care and Use Committee. Prior to euthanasia, mice were anesthetized with sodium pentobarbital (50 mg/kg body weight, intraperitoneal injection) to alleviate pain; euthanasia was then performed via cervical dislocation under deep anesthesia, and tumor tissues were immediately excised upon confirmation of death. Excised tumor tissues were stained with DAPI and imaged for three-channel fluorescence signals, then processed for pathological section preparation and MAF image acquisition following the identical protocol described in the previous paragraph, ensuring procedural consistency with in vitro sample processing.

### Multispectral autofluorescence data acquisition for breast cancer-lung organoid co-culture

The use of human-derived lung tissues for organoid culture was approved under the same ethical clearance as human clinical specimens (Ethics Committee of Chinese PLA General Hospital: S2024-711-01 and S2025-419-01), with written informed consent obtained for all tissue donations. Fresh lung tissues (0.2–0.5 g) were minced into fine fragments using sterile ophthalmic scissors under aseptic conditions, then washed with PBS to remove visible blood clots and necrotic debris. Minced tissues were incubated in 5–10 volumes of collagenase IV solution at 37 °C in a humidified 5% CO₂ atmosphere for 45–60 min; gentle trituration with a 10 mL serological pipette was performed every 15 min to ensure complete dissociation. The resulting cell suspension was filtered through a 70-μm cell strainer to eliminate undigested remnants, and the filtrate was centrifuged at 300 × *g* for 3 min using an Eppendorf 5810R centrifuge. The cell pellet was resuspended in lung organoid medium, mixed with matrigel at a 20:1 (v/v) ratio, and seeded into low-attachment 24-well plates at 50 μL per well. After incubation at 37 °C for 30 min to allow matrigel polymerization, 500 μL of lung organoid medium was added to each well, and organoids were cultured for 7 days prior to co-culture experiments. Cryopreserved MDA-MB-231 breast cancer cells were thawed via gentle agitation in a 37 °C water bath until only ≤2 mm³ ice crystals remained. The cell suspension was immediately diluted in 5 mL of high-glucose Dulbecco’s Modified Eagle Medium supplemented with 5% fetal bovine serum, then centrifuged at 500 × *g* for 3 min to remove cryoprotective agents. The cell pellet was resuspended in breast cancer medium and cultured in T-25 flasks at 37 °C under 5% CO₂. The medium was replaced every 48 h until cells reached 70–80% confluency. For co-culture, MDA-MB-231 cells and lung organoids were harvested, counted using a hemocytometer, and combined at a 5:1 cell ratio. The mixture was seeded into matrigel-precoated low-attachment 24-well plates, and the co-culture system was maintained at 37 °C under 5% CO₂ for 5 days; the medium was replenished every 48 h with a 1:1 mixture of lung organoid medium and breast cancer medium. Post-co-culture, samples were processed following the standard protocol outlined in the previous paragraph to acquire MAF images and H&E images.

### Architecture of virtual staining algorithms

This study aims to transform unlabeled MAF images into H&E-stained style images, a cross-modal image translation task. Since acquiring perfectly paired MAF and H&E images is difficult and costly, we adopt the unsupervised CycleGAN framework, which enables training with unpaired image sets.

**Fig. 7:**
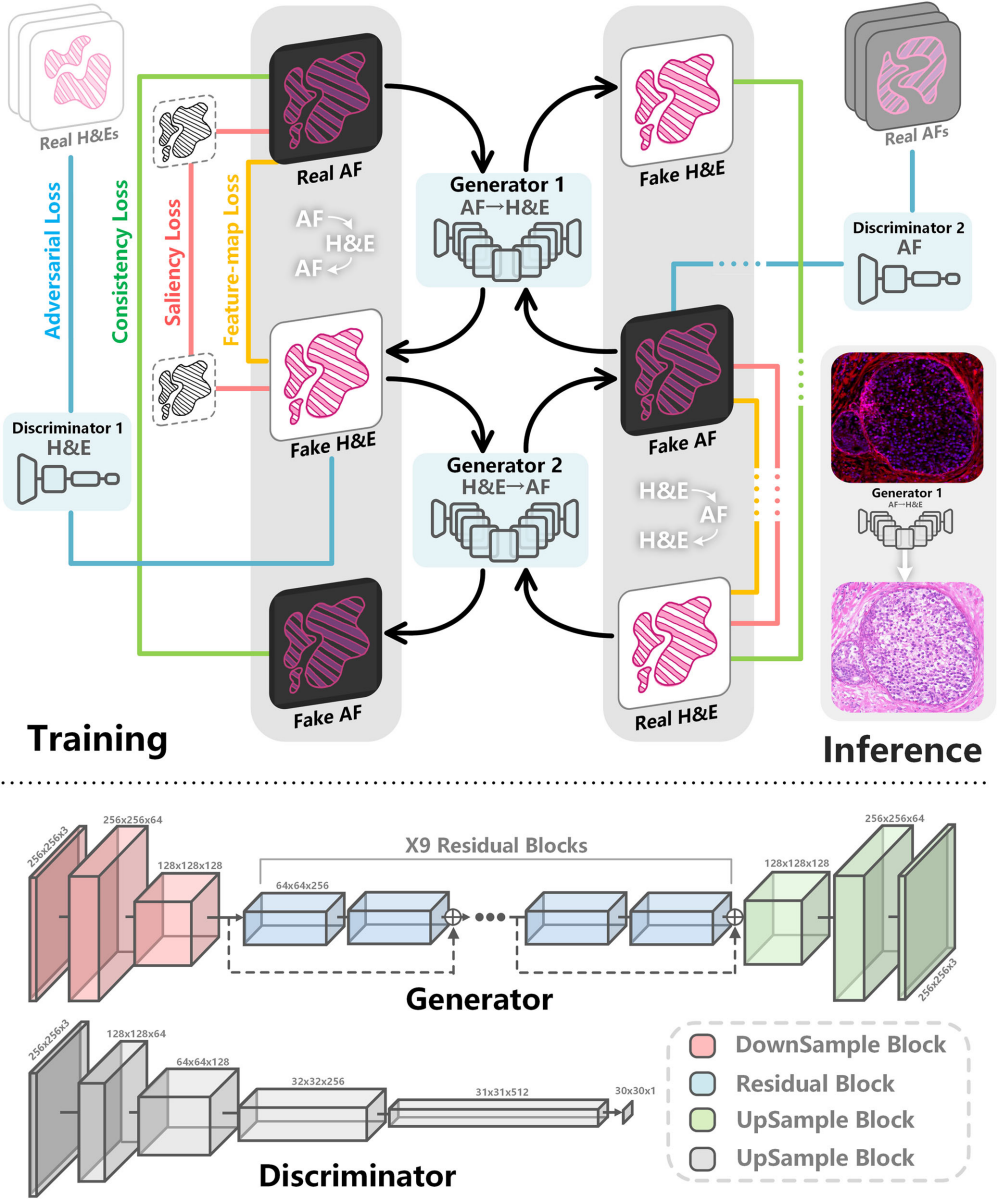
Architecture of the CycleGAN-based virtual staining framework for transforming MAF images to H&E-mimetic histological images, comprising training pipeline, generator/discriminator structures, and inference workflow. Training pipeline (upper panel): The framework employs two reciprocal generators (Generator 1, Generator 2) and discriminators to enable bidirectional translation between real AF images and real H&E images. Generator and discriminator structures (lower panels): The Generator features a modular design with downsample blocks to reduce spatial resolution, residual blocks for feature extraction, and upsample blocks to restore resolution, enabling fine-grained image transformation. The Discriminator utilizes a hierarchical structure to distinguish between real and generated images, providing adversarial feedback to the Generator. The inference workflow (right panel) shows practical deployment: real Autofluorescence (AF) images are input to Generator 1 to produce synthetic H&E images that mimic standard staining, while real H&E images are input to Generator 2 to create synthetic AF images for reverse validation.

Figure 7 shows the virtual staining algorithm architecture we used based on CycleGAN. It mainly consists of the following key components:

#### Bidirectional generators

The system contains two generators. Generator ${G}_{\mathrm{AF}\to \mathrm{HE}}$ converts AF images into vH&E images using an encoder-decoder architecture that extracts semantic features via down-sampling and reconstructs target-style images through up-sampling. Generator ${G}_{\mathrm{HE}\to \mathrm{AF}}$ converts real H&E images into virtual AF images, enforcing “cycle consistency” to preserve content information during translation.

#### Bidirectional discriminators

The system also contains two discriminators. Discriminator ${D}_{\mathrm{HE}}$ used to distinguish real H&E images from vH&E images generated by ${G}_{\mathrm{AF}\to \mathrm{HE}}$, aiming to detect “fake” images. Discriminator ${D}_{\mathrm{AF}}$ used to distinguish real AF images from virtual AF images generated by ${G}_{\mathrm{HE}\to \mathrm{AF}}$.

#### Generator structure

The generator adopts an encoder-decoder design with residual blocks for high-fidelity conversion. The encoder begins with a 7 × 7 convolution mapping inputs to 64 channels, followed by two down-sampling layers that expand channels to 128 and 256 while reducing feature size. At the core, 9 residual blocks transform 256-channel features with skip connections to capture complex mappings and prevent gradient loss. The decoder symmetrically upsamples via two transposed convolutions, reducing channels to 128 and 64, and restores size. A final 7 × 7 convolution outputs the target image with Tanh activation, stabilized by Instance Normalization.

#### Discriminator structure

The discriminator uses a PatchGAN architecture, assessing local rather than global realism. It applies convolutional layers to output an $N\times N$ feature map, where each value indicates the realism of a local region. This local focus compels the generator to produce images with sharp details and realistic textures, reducing blur artifacts. Combined with adversarial training and cycle-consistency loss, the model efficiently learns the mapping from multispectral fluorescence to pathological staining, achieving high-quality virtual staining.

#### Loss functions

The training of the model is driven by four loss functions jointly:

1 $$L={L}_{\mathrm{GAN}}\left({G}_{X\to Y},{D}_{Y},X,Y\right)+{L}_{\mathrm{GAN}}\left({G}_{Y\to X},{D}_{X},Y,X\right)+{{\rm{\lambda }}}_{\mathrm{cyc}}{L}_{\mathrm{cyc}}+{{\rm{\lambda }}}_{\mathrm{feat}}{L}_{\mathrm{feat}}+{{\rm{\lambda }}}_{\mathrm{sal}}{L}_{\mathrm{sal}}$$

#### Adversarial loss

The adversarial loss is the core of GAN and its variants, used to drive the generator to learn the distribution characteristics of the target domain. Specifically, the goal of generator ${G}_{X\to Y}$ is to generate images with the target domain style, while discriminator ${D}_{Y}$ attempts to distinguish whether the input image is a real sample or a fake sample generated by the generator. Its form is:

2 $${L}_{\mathrm{GAN}}\left({G}_{X\to Y},{D}_{Y},X,Y\right)={E}_{y\sim {p}_{\mathrm{data}}\left(y\right)}\left[\log {D}_{Y}\left(y\right)\right]+{E}_{x\sim {p}_{\mathrm{data}}\left(x\right)}\left[\log \left(1-{D}_{Y}\left({G}_{X\to Y}\left(x\right)\right)\right)\right]$$

Similarly, generator ${G}_{Y\to X}$ and discriminator ${D}_{X}$ also have corresponding loss functions. Through this adversarial mechanism between the generator and the discriminator, the model can gradually improve the authenticity of the generated results and the style matching degree.

#### Cycle-consistency loss

Since training data often lack paired samples, CycleGAN guarantees the reversibility of cross-domain mapping through the cycle consistency constraint. That is, an image mapped from source domain $X$ to target domain $Y$, and then mapped back to $X$ by the reverse generator, should be as close as possible to the original input:

3 $${L}_{\mathrm{cyc}}\left({G}_{X\to Y},{G}_{Y\to X}\right)={E}_{x\sim {p}_{\mathrm{data}}\left(x\right)}\left[\Vert {G}_{Y\to X}\left({G}_{X\to Y}\left(x\right)\right)-x{\Vert }_{1}\right]+{E}_{y\sim {p}_{\mathrm{data}}\left(y\right)}\left[\Vert \left({G}_{Y\to X}\left(y\right)\right)-y{\Vert }_{1}\right]$$

This constraint can effectively prevent the generator from learning “meaningless transformations,” so that the translated image maintains semantic consistency with the input.

#### Auxiliary content preservation constraints

To address the common problems of content drift and detail loss in unsupervised translation, we introduce the following two regularization constraints:

#### Global feature consistency loss

Pixel-space constraints alone may not ensure structural preservation. Thus, we introduce a global feature consistency loss, using a VGG16 network pretrained on ImageNet to compute perceptual loss. Feature maps from layers relu1_1, relu2_1, relu3_1, relu4_1, and relu5_1 were extracted using Vgg16 Experimental model. The L1 distance between these features of real and generated images enforced global color and structural consistency:

4 $${L}_{\mathrm{feat}}=\frac{1}{|{L}_{\mathrm{feat}}|}\mathop{\sum }\limits_{l\in {L}_{\mathrm{feat}}}\frac{1}{{C}_{l}{H}_{l}{W}_{l}}\left({E}_{x\sim {p}_{\mathrm{data}}\left(x\right)}\left[{||{V}_{l}^{\mathrm{pt}}\left(x\right)-{V}_{l}^{\mathrm{pt}}\left({G}_{X\to Y}\left(x\right)\right)|}_{2}^{2}\right]+{E}_{y\sim {p}_{\mathrm{data}}\left(y\right)}\left[|{V}_{l}^{\mathrm{pt}}\left(y\right)-{V}_{l}^{\mathrm{pt}}\left({G}_{Y\to X}\left(y\right)\right){|}_{2}^{2}\right]\right)$$

Here, ${{\rm{L}}}_{\mathrm{feat}}$ is the set of feature layers used for perceptual alignment, ${V}_{l}^{\mathrm{pt}}\left(\cdot \right)$ is the feature mapping at layer $l$ extracted by the pretrained feature extractor, and ${C}_{l},{H}_{l},{W}_{l}$ are the channel number and spatial dimensions of the feature map at layer $l$, used for normalization.

#### Saliency consistency loss

This constraint enhances the model’s ability to preserve key regions such as nuclei and lesion boundaries. Specifically, saliency maps are generated for both AF and vH&E images using grayscale thresholding, highlighting important structures like nuclei. Saliency maps were generated by converting images to grayscale and applying fixed thresholding (value = 170), followed by 3 × 3 morphological opening and closing to remove small noise and preserve nuclei boundaries. The L1 loss between these maps guides the model to maintain nuclei morphology and distribution during virtual staining, improving biological fidelity and diagnostic value.

5 $${L}_{\mathrm{sal}}={E}_{x\sim {p}_{\mathrm{data}}\left(x\right)}\left[{{||}{T}_{\alpha }\left(a\right)-{T}_{\beta }\left({G}_{X\to Y}\left(x\right)\right){||}}_{1}\right]+{E}_{y\sim {p}_{\mathrm{data}}\left(y\right)}\left[{{||}{T}_{\beta }\left(b\right)-{T}_{\alpha }\left({G}_{Y\to X}\left(y\right)\right){||}}_{1}\right]$$

Here, ${T}_{\alpha },{T}_{\beta }$ are the operators for extracting saliency maps from domain $X$ and domain $Y$ images.

### Training details MAF/H&E breast cancer histopathology image translation

#### Data preparation

We collected 30 whole-slide images of breast cancer tissues. Through segmentation and background filtering algorithms, we generated approximately 400,000 unpaired image patches of 256 × 256 pixels, which form our open-source dataset contribution. Due to computational training constraints, we utilized 10% of these patches for model development, including 19,989 MAF patches and 22,778 H&E patches. The dataset was partitioned such that 10% served as the validation set, and the remainder was used for training. Standard augmentation methods, such as random flipping and rotation, were applied during training. The model is implemented based on the PyTorch framework and trained on NVIDIA RTX 3090 GPUs. We use the Adam optimizer with an initial learning rate set to 0.0002 and a batch size of 4. The model is trained for a total of 200 cycles (epochs), where the learning rate is kept constant for the first 100 cycles, and the learning rate is linearly decayed to zero for the next 100 cycles to ensure full convergence of the model.

In order to objectively assess the authenticity and diversity of the virtual coloring results, this study adopts the FID and the KID as quantitative metrics. Both are based on the high-level feature space of the Inception-V3 network to compare the distribution of the generated image with that of the real image. FID assumes that the feature distribution approximates a Gaussian distribution, and measures the difference between the two by calculating the Fréchet Distance between the mean and the covariance, with smaller values indicating that the generated image is closer to the real image in terms of the overall distribution; while KID is based on the MMD, with a polynomial kernel, and is used as a quantitative index. MMD, which compares distributions under a polynomial kernel, does not require Gaussian assumptions and is more stable under small sample conditions. In the experiments, we scaled all the images to 299 × 299 uniformly and extracted 2048-dimensional features from the Inception-V3 pool3 layer after normalizing to [0,1], and evaluated them on 456 test images. Each group of experiments was calculated independently 5 times and averaged. The final FID and KID values can effectively reflect the structure restoration and detail reconstruction of the virtual stained images and the real H&E-stained images. The final FID and KID values can effectively reflect the proximity between the virtual stained image and the real H&E-stained image in terms of structure restoration and detail reconstruction.

### Ethical approval

The study was conducted in accordance with the Declaration of Helsinki and approved by the Ethics Committee of Chinese PLA General Hospital (protocol code S2024-711-01 and date of approval: November 28, 2024; S2025-419-01 and date of approval: May 29, 2025) for studies involving humans. It was also approved by the Ethics Committee of Beijing Viewsolid Biotechnology Co. LTD (protocol code VS2126A00750 and date of approval: February 1, 2025) for studies involving animals.

## Supplementary information

Supplementary_Materials

## Data Availability

The datasets generated and analyzed during the current study are available in the Zenodo repository, Ye, J., & Sun, J. (2025). 301 AF2HE datasets [Dataset]. Zenodo. https://zenodo.org/records/17646116. All other data and materials related to this study are available from the corresponding author on reasonable request.
