# Supplementary material for: Artificial intelligence assisted multi-model pathological diagnosis of breast cancer based on multispectral autofluorescence images
Source: NPJ Breast Cancer. 2026 Mar 12;12:62. doi: 10.1038/s41523-026-00915-2 (PMC13121592; doi:10.1038/s41523-026-00915-2)
Supplement: Supplementary file 1 — Supplementary_Materials [file 41523_2026_915_MOESM1_ESM.pdf]

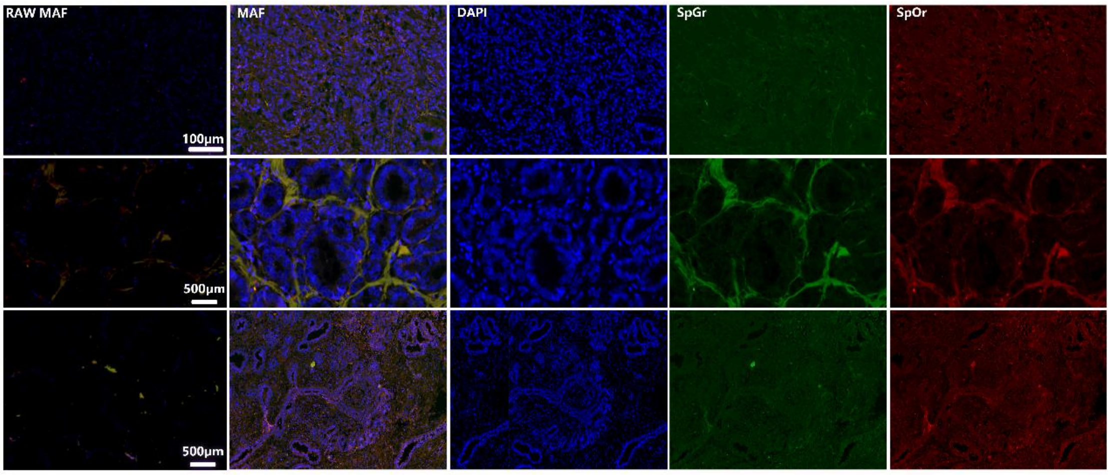

Figure S1. Raw and contrast-adjusted MAF images of unstained tissue sections. First column (RAW MAF): shows the original, unmodified MAF images acquired from unstained tissue sections. Columns 2–5 (MAF, DAPI, SpGr, SpOr): Present composite MAF images and their decomposed spectral channels. These images underwent non-destructive contrast adjustment (modifying Blank and Gamma parameters via CaseViewer software) solely to enhance the visibility of cellular structures for illustrative clarity, consistent with the post-processing applied to images in the manuscript (e.g., Fig. 1, Fig. 4).

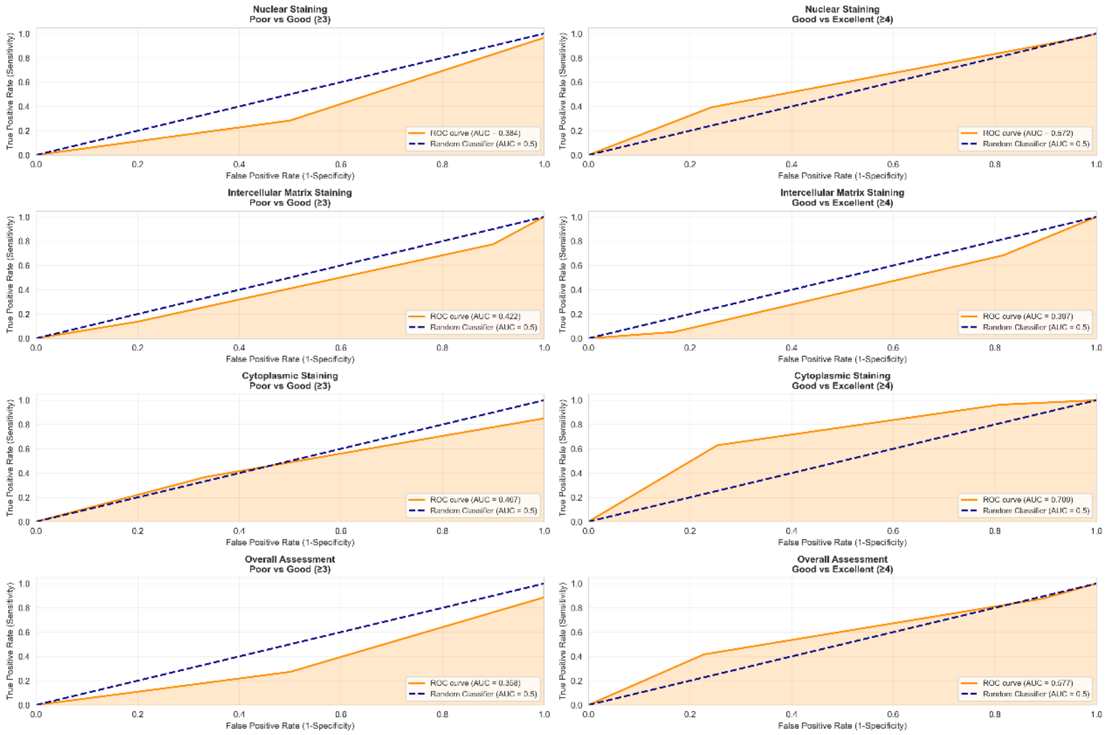

Figure S2. Receiver Operating Characteristic (ROC) Curves for Distinguishing vH&E from Standard H&E Staining.Each subplot assesses the ability to distinguish virtual H&E (vH&E) from standard H&E staining across four histologic features and two clinical scenarios:Rows: Histologic features (from top to bottom: nuclear staining details, intercellular matrix staining details, cytoplasmic staining details, overall staining assessment);Columns: Clinical scenarios (left: "Poor vs Good" with composite score ≥ 3 defined as "Good"; right: "Good vs Excellent" with composite score ≥ 4 defined as "Excellent").The orange curves represent ROC curves for vH&E, and blue dashed lines represent random classifiers (AUC = 0.5). All AUC values are close to 0.5, demonstrating that vH&E staining is statistically indistinguishable from standard H&E staining—confirming their clinical equivalence.

SUPPLEMENTARY TABLE SI

WILCOXON SIGNED-RANK TEST RESULTS FOR VIRTUAL VS. STANDARD HISTOLOGICAL STAINING ACROSS MORPHOLOGICAL FEATURES

Feature Category	n	Mean ± SD (vHE；standard HE)	Median	Negative Rank Sum	Positive Rank Sum	W Statistic	Z Statistic	p-value
Nuclear Staining	90	3.26 ± 0.51; 3.29 ± 0.50	3.00	289.50	340.50	289.50	-0.467	0.640
Cytoplasmic Staining	90	2.93 ± 0.60; 3.10 ± 0.56	3.00	448.00	777.00	448.00	-1.768	0.077
Intercellular Matrix Staining	90	3.22 ± 0.68; 3.27 ± 0.51	3.00	371.00	449.00	371.00	-0.588	0.556
Overall Staining	90	3.17 ± 0.60; 3.24 ± 0.48	3.00	290.50	412.50	290.50	-1.010	0.312

SUPPLEMENTARY TABLE SII

COMPREHENSIVE SCORING RUBRIC

Grade	Scoring Rubric
1	Unacceptable: Key structural details invisible, non-diagnostic
2	Acceptable: Basic structures visible but details blurred, marginally diagnostic
3	Good: Clear structural details, fully meeting diagnostic requirements
4	Excellent: Sharp structural contrast, optimal for fine-grained pathological assessment

SUPPLEMENTARY TABLE SIII

NUCLEAR STAINING DETAIL SCORING CONFUSION MATRIX

STANDARD H&E / VH&E	2	3	4
2	15 (8.3%)	10 (5.6%)	0 (0.0%)
3	6 (3.3%)	102 (56.7%)	12 (6.7%)
4	0 (0.0%)	18 (10.0%)	9 (5.0%)

SUPPLEMENTARY TABLE SIV

CYTOPLASMIC STAINING DETAIL SCORING CONFUSION MATRIX

STANDARD H&E / VH&E	2	3	4
2	12 (6.7%)	15 (8.3%)	0 (0.0%)
3	10 (5.6%)	90 (50.0%)	18 (10.0%)
4	0 (0.0%)	22 (12.2%)	8 (4.4%)

SUPPLEMENTARY TABLE SV

INTERCELLULAR MATRIX STAINING DETAIL SCORING CONFUSION MATRIX

STANDARD H&E / VH&E	2	3	4
2	14 (7.8%)	12 (6.7%)	0 (0.0%)
3	9 (5.0%)	93 (51.7%)	15 (8.3%)
4	0 (0.0%)	20 (11.1%)	7 (3.9%)

SUPPLEMENTARY TABLE SVI

OVERALL STAINING DETAIL SCORING CONFUSION MATRIX

STANDARD H&E / VH&E	2	3	4
2	18 (10.0%)	12 (6.7%)	0 (0.0%)
3	8 (4.4%)	95 (52.8%)	15 (8.3%)
4	0 (0.0%)	17 (9.4%)	10 (5.6%)

SUPPLEMENTARY TABLE SVII

CORE DATA OF ROC CURVE ANALYSIS

(QUANTIFIES THE CLINICAL DISTINGUISHABILITY BETWEEN VH&E AND STANDARD H&E)

Analysis Scenario	Core Statistical Metrics	Value
Scenario 1: Poor vs Good (Composite score ≥ 3 = Good; < 3 = Poor)	AUC	0.463
	Sensitivity	0.872
	Specificity	0.059
Scenario 2: Good vs Excellent (Composite score ≥ 4 = Excellent; 2 ≤ score < 4 = Good)	AUC	0.584
	Sensitivity	0.398
	Specificity	0.779
